# Targeted Therapy as a Potential De-Escalation Strategy in Locally Advanced HPV-Associated Oropharyngeal Cancer: A Literature Review

**DOI:** 10.3389/fonc.2021.730412

**Published:** 2021-08-17

**Authors:** Lennox Chitsike, Penelope J. Duerksen-Hughes

**Affiliations:** Department of Basic Sciences, Loma Linda University School of Medicine, Loma Linda, CA, United States

**Keywords:** HPV, HNSCC, OPSCC, de-escalation, targeted therapies, radiation, sensitization, adverse effects

## Abstract

The treatment landscape of locally advanced HPV-oropharyngeal squamous cell carcinoma (OPSCC) is undergoing transformation. This is because the high cures rates observed in OPSCC are paired with severe treatment-related, long-term toxicities. These significant adverse effects have led some to conclude that the current standard of care is over-treating patients, and that de-intensifying the regimens may achieve comparable survival outcomes with lower toxicities. Consequently, several de-escalation approaches involving locally advanced OPSCC are underway. These include the reduction of dosage and volume of intensive cytotoxic regimens, as well as elimination of invasive surgical procedures. Such de-intensifying treatments have the potential to achieve efficacy and concurrently alleviate morbidity. Targeted therapies, given their overall safer toxicity profiles, also make excellent candidates for de-escalation, either alone or alongside standard treatments. However, their role in these endeavors is currently limited, because few targeted therapies are currently in clinical use for head and neck cancers. Unfortunately, cetuximab, the only FDA-approved targeted therapy, has shown inferior outcomes when paired with radiation as compared to cisplatin, the standard radio-sensitizer, in recent de-escalation trials. These findings indicate the need for a better understanding of OPSCC biology in the design of rational therapeutic strategies and the development of novel, OPSCC-targeted therapies that are safe and can improve the therapeutic index of standard therapies. In this review, we summarize ongoing research on mechanism-based inhibitors in OPSCC, beginning with the salient molecular features that modulate tumorigenic processes and response, then exploring pharmacological inhibition and pre-clinical validation studies of candidate targeted agents, and finally, summarizing the progression of those candidates in the clinic.

## Introduction

Head and neck squamous cell carcinomas (HNSCCs) arise from anatomical regions of the head and neck that include the oral cavity, oropharynx, hypopharynx, larynx, and nasopharynx (1). Although all HNSCCs originate from transformed keratinocytes, and had for many years remained largely undifferentiated, two distinct subtypes have recently been recognized. One subtype (HPV^+^-OPSCC) is causally associated with the presence of the human papilloma virus (HPV), and predominantly forms in the oropharynx (OPSCC). The other subtype (HPV^–^HNSCC) is caused primarily by alcohol and tobacco usage, and is more anatomically distributed. Epidemiologically, HPV^+^-OPSCC has been rising substantially in the past 4 decades. In parallel, HPV^–^HNSCC has been declining and shifting in the opposite direction (1, 2). The demographics are also different; HPV^+^-OPSCC tends to affect younger and otherwise healthier people than does HPV^–^HNSCC (3–5). More importantly, across all treatment modalities, HPV^+^-OPSCC patients have greater response to treatment and substantially better locoregional control and survival outcomes (3, 6). One of first key studies to report this distinct clinical behavior of the HNSCC subtypes was conducted Ang KK et al. (7). They reported that the 3-year overall survival (OS) for stage III or IV patients treated with chemoradiation was 82.4% in the HPV^+^ patients versus 57.1% in the HPV^-^ subgroup (7). A longer-term study by Nguyen-Tan PF et al. confirmed these findings and found that 8-year rates were 70.9 versus 30.2% for OS, 64.0 versus 23.3% for progression free survival, 19.5 versus 52.4% for loco- regional failure, and 10.3 versus 16.1% for distant metastases in p16^+^ (HPV positive status) OPSCC versus p16^-^ negative (HPV negative status) patients (8). This prognostic significance has motivated recent changes in the latest edition (8^th^) of The American Joint Committee on Cancer (AJCC) staging system, which downgrades the Tumor, Nodes, Metastasis (TNM) classification of HPV^+^-OPSCC relative to HPV-HNSCC (1, 6). Despite the TNM reclassification, the treatment guidelines for OPSCC as governed by The National Comprehensive Cancer Network Clinical Practice Guidelines in Oncology (NCCN) have not changed (2, 9). Essentially, there is still no discrimination between HNSCC subtypes by p16 status; radiotherapy or surgery are recommended for early-stage disease and combinatorial therapy of surgery and adjunct radiotherapy or concomitant chemoradiation therapy (CRT) are used for malignant disease (9). Unfortunately, use of these standard treatments is toxic to the delicate tissue and muscles surrounding organs of the head and neck. The majority of patients present with locally advanced (LA) disease and definitive radiotherapy (RT) using a standard dose of 70 Gy causes long-term morbidities such as dysphagia, xerostomia, ototoxicity, soft tissue fibrosis, trismus, and other conditions. and this is often exacerbated by cisplatin, the current standard radio-sensitizer (1, 9). Many now believe that the standard regimens are excessive for the HPV^+^ subtype and should be de-escalated in the LA, curative intent setting. The overall goal of this de-escalation is to lower the toxicity and improve the quality of life without compromising current and favorable survival outcomes for patients with HPV^+^-OPSCC.

**Figure 1:**
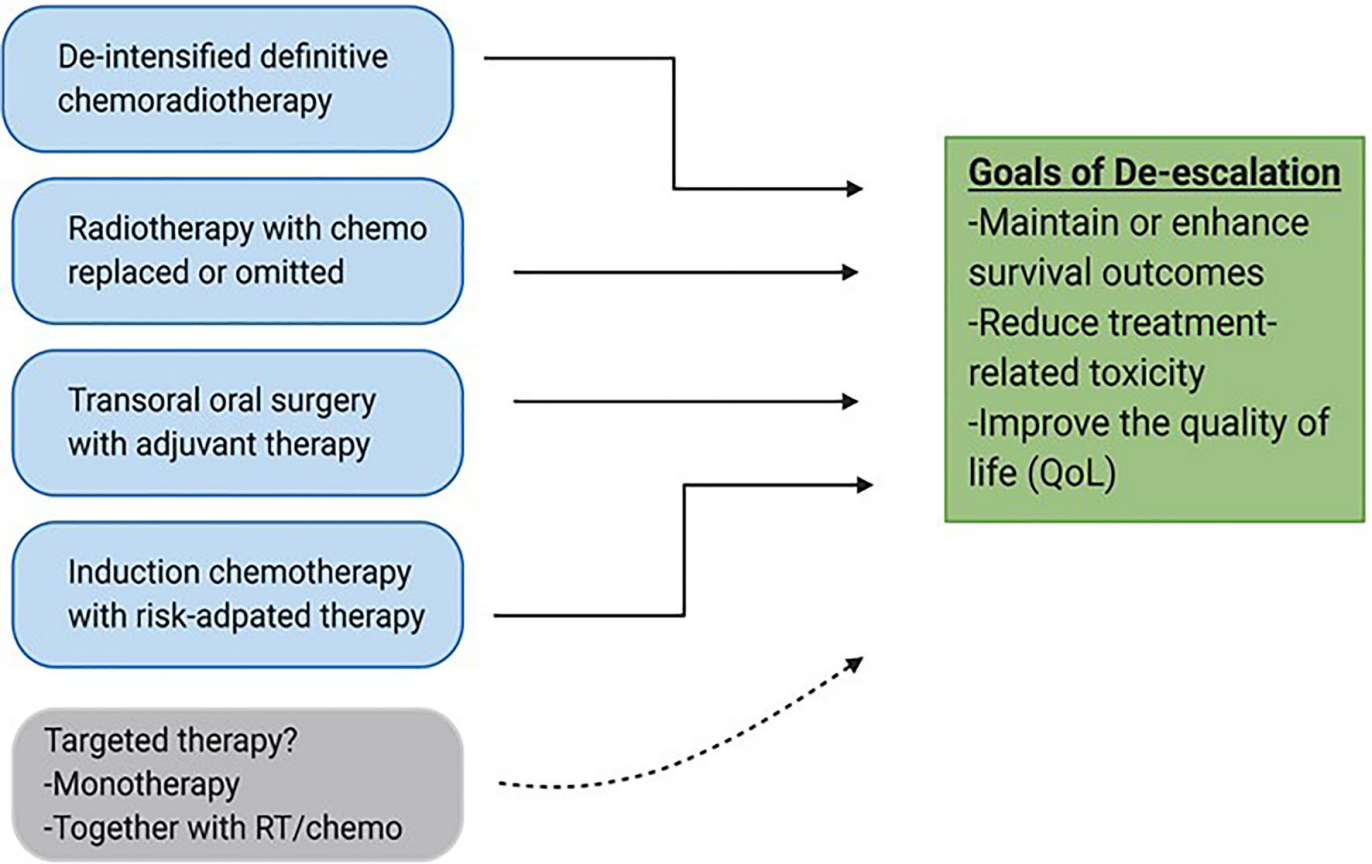
De-escalation strategies for HPV-associated OPSCC. Various approaches are currently being pursued to reduce volumes, dosages and invasiveness of current standard treatments with the goal of maintaining survival outcomes and improving safety and quality of life (QoL). Targeted therapy has the potential to be integrated into de-escalated regimens especially together with conventional treatments such as radiation.

For these reasons, a separate therapeutic strategy is needed for the HPV^+^ patient cohort. To this end, a number of de-escalation strategies are currently being pursued. These strategies generally include reduction of target volume, dosages of RT and chemotherapy, reduction of the number of modalities in combinatorial regimens, radiation treatment guided by response to induction chemotherapy, and use of minimally invasive surgery as shown in **Figure 1** (3). Early results from most of these approaches have so far been encouraging, and results from longer follow-up studies are now awaited. The hope is that these longer-term studies will support clinical adoption of new and less toxic regimens. Another therapeutic strategy under study is the de-intensification of chemoradiotherapy by substituting cisplatin with a less toxic systemic alternative, cetuximab. The expectations of this move were fairly high given that cetuximab, an anti-EGFR biologic, is relatively tolerable as a targeting agent and is already approved in combination with radiation, particularly for patients who cannot tolerate cisplatin (5, 10, 11). Recent data from de-intensification trials, however, has shown that cetuximab is inferior to cisplatin, and is therefore not recommended for definitive treatment (10–12). This issue is further compounded by the fact that there are no additional targeting agents currently in use for HNSCC that could readily take the place of cisplatin in these de-intensified regimens. Finding such additional selective and safer targeting therapies that can be used effectively as monotherapies or synergistically together with standard therapies is therefore of paramount importance. In this review, we discuss differentially regulated genetic and molecular features in HNSCC that can be exploited for targeted therapy, with a special emphasis on HPV^+^-HNSCC. We then highlight pre-clinical research done to validate the functional importance of these unique alterations. Finally, we briefly review the translational relevance of targetable alterations by looking at impact and progress of targeted agents currently in clinical studies and their potential utility as agents for de-escalation in the future.

## Targetable Alterations in HNSCC

The success of targeted therapy in many solid tumors has been propelled by research that elucidates genomic events and molecular alterations driving the growth, survival, differentiation, angiogenesis, and migration of tumors. In HNSCC, changes in the genetic landscape and the resulting aberrant signaling have recently been catalogued through various next generation sequencing (NGS) tools, setting the stage for mechanism-driven research (4, 13–16). NGS analyses show that HPV^-^ and HPV^+^ cancers are genetically distinct and heterogeneous diseases. The most dominant mutations in HPV^–^HNSCC affect cell cycle control and involve inactivation of the p53 and Rb-p16INK4A–cyclin D1 pathways (14, 15). In addition, genomic events of significance in HPV^–^HNSCC have also been identified for the MAPK, Wnt, Notch and Myc genes. In terms of tyrosine kinase oncogenes, mutations and alterations are significant for EGFR, FGFR1 and c-Met (14, 15, 17, 18). Other mutations in cancer-associated genes have been found, but their frequency is low, which puts their driver assignment in question. HPV^+^-OPSCC, on the other hand, exhibits fewer genetic alterations in tumor suppressor genes, and more in the genes that govern the PI3K pathway. To a lesser extent, the NFkB and JAK/STAT pathways and genes encoding BRCA1/2 and Kras have also been found to be altered (4, 14–16). For tyrosine kinases, activating mutations have been recently confirmed for FGFR2/3 (14–16). As in HPV^–^HNSCC, most of the remaining genes have mutations at low frequencies.

Collectively, the number and diversity of genetic alterations in cancer-relevant genes found in HPV^+^-HNSCC (and HPV-unrelated HNSCC) are comparatively fewer than in other cancers. In principle, this limits the number of druggable targets and consequently, the respective translational efforts aimed at improving patient outcomes in HNSCC. That said, the integration of the HPV genome in transformed keratinocytes and the continuous expression of associated viral proteins in HPV^+^ tumors may provide additional molecular and biological alterations of therapeutic relevance. Not only do the HPV proteins influence the global methylation profile of HPV^+^-tumor cells and in turn their own expression, studies show that they modulate the expression of other cellular oncogenes including members of the ErBb/Her family (19–21). HPV oncoproteins are also known to derange the DNA damage response (DDR) of host cells by compromising the function of key proteins that are necessary to detect and repair the effects of DNA damaging agents (3). In addition, the two HPV oncoproteins E6 and E7 directly promote cell proliferation and interfere with the normal apoptotic circuitry by eliminating the proteins that regulate mitosis and host cell death (3, 22). Moreover, HPV-derived proteins affect the immunological anti-tumor response, making the tumor microenvironment of HPV^+^-OPSCC quite different from that seen in other cancers (3). Thus, it is feasible to leverage the unique biology of HPV^+^-OPSCC for the rational development of agents that can efficaciously and safely ablate tumor cells.

## Targeting the PI3K Signaling and Pathway

**Figure 2:**
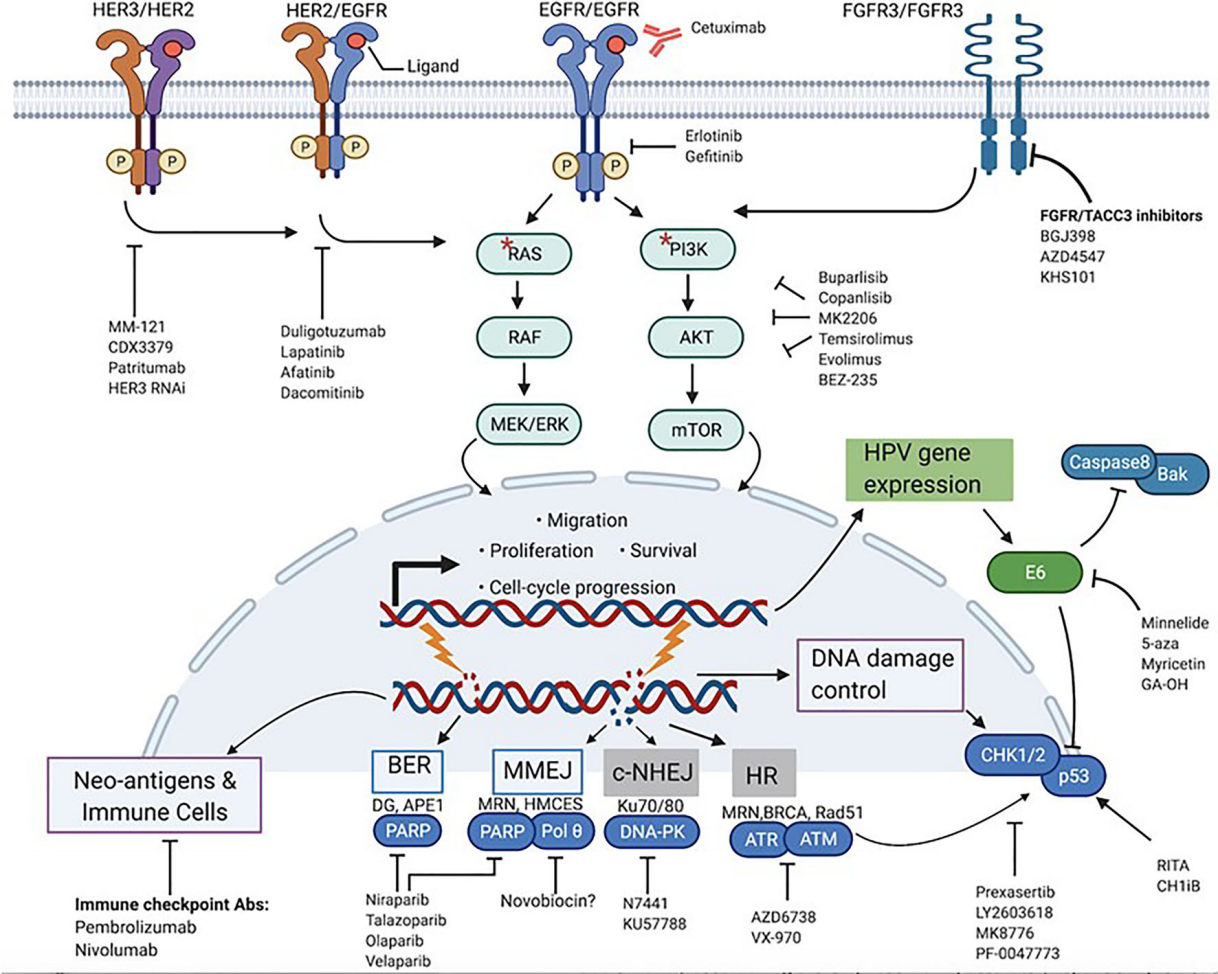
Actionable targets for mechanism-based inhibition in HPV^+^-HNSCC. Prominent protein targets present at the membrane, cytoplasm and nuclear level that play various cancer-promoting roles in HPV^+^-HNSCC and their associated signaling pathways are highlighted. Respective target-specific and pathway-directed small molecules and biologic agents designed to stop or slow down growth and survival of cancer cells are shown. * represents mutations in RAS and PI3K.

The PI3K pathway is one of the most potent mitogenic signaling pathways in cancer, governing cellular processes ranging from proliferation to survival, metabolism, differentiation, and invasion (23, 24). PI3Ks are lipid membrane enzymes that can be subdivided into 3 classes: class I, II, III. Of these 3 classes, class IA is the most implicated in cancer, and comprises heterodimeric isoforms of the p110 catalytic subunit (encoded by the PI3KC) and the p85 regulatory subunit (encoded by the PI3KR). Binding of growth factor ligands and subsequent autophosphorylation of receptor tyrosine kinases (RTKs) such as ErBb/Her receptors initiates the signaling cascade of the PI3K pathway (23–26). Once phosphorylated, RTKs will bind and activate PI3Ks, which in turn are responsible for producing the second messenger phosphatidyl-inositol-3,4,5 triphosphate (PIP_3_). This phosphorylated lipid will then transduce the signal to major downstream effectors of the PI3K pathway, Akt and mTOR, to modulate processes that are key to the growth and metastasis of many cancers as shown in **Figure 2** (23–26). Not surprisingly, the PI3K/Akt/mTOR pathway is the most frequently dys-regulated pathway in HNSCC, more than other mitogenic pathways such as MAPK/Erk and JAK/Stat (1, 26). In the PI3K pathway, the PI3KCA gene is the component that is by far the most commonly altered, with frequencies of up to 56% in HPV^+^- and 34% in HPV^–^HNSCC tumors (15, 25, 26). Mutations in other components of the PI3K pathway such as PTEN and PI3KR genes also show bias towards HPV^+^-OPSCC, but at significantly lower rates than does PI3KCA (25). Interestingly, the location of hotspot mutations in the PI3KCA is also different. In HPV^+^-tumors, the mutations are predominantly found in the helical domain of PI3K while in their HPV-counterparts, the mutations are more spread out in the helical and kinase domains (1, 15). Generally, these PI3K mutations are activating and are associated with hyperstimulation of downstream factors, making targeting of the effectors of this pathway potentially attractive.

### PI3K/Akt/mTOR as Targets

Numerous PI3K pathway inhibitors are on the market and have already been tested in HNSCC clinically, including in locally advanced HNSCC (**Figure 2** and **Table 1**). These include PI3K isoform-selective, pan-PI3K, Akt, mTOR and dual PI3K/mTOR inhibitors (15, 16, 25–27). Broadly speaking, the results from clinical evaluation of most of these inhibitors thus far have been underwhelming, highlighted by low objective responses, limited gains in survival and/or intolerable toxicities (28). Prime examples include mTOR inhibitors such as everolimus and temsirolimus, and the P13K inhibitors PX-866, copanlisib and dactolisib. The only inhibitor targeting the PI3K/Akt/mTOR pathway that has so far shown survival benefits is buparlisib (29), as buparlisib in combination with paclitaxel did demonstrate an improvement in both PFS and OS (29). Interestingly, a subgroup analysis showed that patients with HPV-positivity did not benefit from this treatment (30). This is rather surprising, considering that the rationale for PI3K inhibition, based on genomic studies, seemed stronger for the HPV^+^ subtype. In contrast, some pre-clinical studies using PDX models have demonstrated that HPV^+^ tumors with PI3K activating mutations can be effectively controlled using PI3K or PI3K/mTOR inhibitors (31, 32). Similarly, mTOR inhibitors were also shown to be effective in inhibiting the growth of HPV^+^ PDX tumors (33, 34). Can these conflicting results be reconciled? Possibly. One observation that may help to explain this discrepancy is that even though some studies have shown functional consequences of relevant PI3KCA mutations in OPSCC, pre-clinical validation has not been extensive. This is because many commercially available HPV^+^ cell lines do not carry the mutations seen clinically, which limits the information gained from *in vitro* research (31). Also, some studies have shown that the correlation between PI3K activation and inhibitor sensitivity is weak, as activity of PI3K inhibitors was similar in cells expressing wildtype or mutant PI3KCA (25, 35). In fact, one study showed that introducing PI3KCA hotspot mutations (E545K or H1047R) to cells actually decreased their sensitivity to PI3K inhibitors (25, 36). Only when dual inhibitors were used was this behavior reversed. In addition, it was also revealed that the H1047R mutation was more sensitive to inhibition than was the E545K mutation. This is intriguing, considering that PI3KCA mutations in HPV^+^ cancers are predominantly helical and that HPV^-^ tumors have more kinase domain mutations (H1047R). Studies using transgenic mice and cancer cells have shown that the two types of mutants activate their effectors through different mechanisms and that the H1047R substitution may be the better activator of Akt (37–39). Conversely, a study in HPV^+^-OPSCC showed that PI3K signaling through PI3KCA mutations activated mTOR but not Akt, and that this signaling was more sensitive to PI3K/mTOR dual inhibition than to Akt inhibition (40). More importantly, not only were helical domain mutations associated with poorer clinical prognosis compared to kinase domain mutations in breast cancer, a clinical study found that helical domain mutations also had lower response rates to PI3K/Akt/mTOR inhibitors (41, 42). In fact, some studies have shown that HPV^+^ cancer cells have intrinsic resistance towards PI3K inhibition (25, 43). These observations certainly highlight the complexity of PI3K signaling in HNSCC and the need for further research.

**Table 1:** Summary of past and ongoing clinical trials evaluating targeted agents alone or in combination with standard treatments specifically in disease conditions that involve locally advanced HNSCC and/or HPV-associated OPSCC.

Target	Agent	Additional Therapy	Disease condition	Trial Identifier	Phase	Status
PI3K	PX-866	Docetaxel	Locally advanced or RM HNSCC	NCT01204099	2	Completed
	Alpelisib (BYL719)	Cisplatin, IMRT	Locally advanced HNSCC	NCT02537223	1	Completed
		Cetuximab, cisplatin	Locally advanced, HPV+ OPSCC	NCT02298595	2	Withdrawn
		Cetuximab, IMRT	Locally advanced HNSCC	NCT02282371	1	Active, not recruiting
		Conventional surgery	Stage I-IVA HPV+ OPSCC	NCT03601507	2	Recruiting
	Buparlisib	Cisplatin, IMRT	Locally advanced HNSCC	NCT02113878	1	Active, not recruiting
	Eganelisib (IPI-549)	–	Locally advanced HNSCC	NCT03795610	2	Recruiting
MTOR	Everolimus	–	Locally advanced or RM HNSCC	NCT01051791	2	Terminated
		–	Locally advanced HNSCC	NCT01133678	2	Terminated
		Carboplatin, Radiation	Locally advanced HNSCC	NCT01333085	2	Completed
		Cisplatin, IMRT	Locally advanced HNSCC	NCT01058408	1	Terminated
		–	Locally advanced HNSCC	NCT01111058	2	Terminated
		–	Locally advanced HNSCC	NCT00935961	1	Completed
PARP	Olaparib	Cetuximab, Radiation	Locally advanced HNSCC	NCT01758731	1	Completed
		–	Surgically resectable HNSCC	NCT02686008	1	Withdrawn
		Cisplatin, IMRT	Locally advanced HNSCC	NCT01491139	1	Withdrawn
		Cisplatin, IMRT	Locally advanced HNSCC	NCT02308072	1	Active, not recruiting
	Velaparib	Cisplatin,carboplatin, paclitaxel, 5-FU	Stage IVA/B HNSCC	NCT01711541	2	Active, not recruiting
DNA-PK	Peposertib	IMRT	Locally advanced HNSCC	NCT04533750	1	Recruiting
CHK1/2	Prexasertib	Cisplatin, Cetuximab, IMRT	Locally advanced HNSCC	NCT02555644	1	Completed
Wee1	AZD1775	Cisplatin, IMRT	Locally advanced HNSCC	NCT02585973	1	Completed
	MK-1775	Cisplatin, Docetaxel, surgery	Locally advanced HNSCC	NCT02508246	1	Completed
DNA Methylase	5-azacytidine	–	Surgically resectable HNSCC	NCT02178072	2	Recruiting
Immune Checkpoints	Nivolumab	Cisplatin, Radiation	Stage I-II HPV+ OPSCC	NCT03952585	2/3	Recruiting
		Cisplatin, IMRT	Stage II-III HPV^+^ OPSCC	NCT03811015	2/3	Recruiting
		Multiple including cisplatin, TORS,IMRT	Locally advanced HPV^+^ OPSCC	NCT03107182	2	Active, not recruiting
		Radiation	Locally advanced HPV+ OPSCC	NCT03715946	2	Active, not recruiting
	Durvalumab, Tremelimumab	SBRT, Surgery	Stage I-III HPV^+^ OPSCC	NCT03618134	2	Recruiting
	Ipilimumab, Nivolumab	IMRT	Stage I-IVA HPV+ OPSCC	NCT03799445	2	Recruiting
	Durvalumab, Tremelimumab	Radiation	Locally advanced HPV^+^ OPSCC	NCT03410615	2	Recruiting
	Pembrolizumab	Cisplatin, Radiation	Locally advanced HPV^+^ OPSCC	NCT03383094	2	Recruiting

5-FU, 5 flourouracil; IMRT, intensity modulated radiation therapy; R/M, recurrent/metastasis; SBRT, Stereotactic Body Radiation Therapy; TORS, Transoral surgery.

### RTKs as Targets

As alluded to above, PI3K signaling is instigated by activation of RTKs (**Figure 2**). In addition, RTKs may be involved in compensatory activation in response to PI3K inhibitors. This makes inhibition of activated RTKs alone or in combination with P13K inhibitors a potentially effective therapeutic strategy. Developing research shows that one such RTK is the FGFR. A recent study has implicated the FGF pathway as a viable target in HPV^+^-OPSCC (32). Activating alterations in FGFR2/FGFR3 including FGFR3–TACC3 fusions were found in 17.6% of HPV-positive tumors. Furthermore, FGFR3 alterations have previously been found to mediate resistance to EGFR inhibition. Therapeutically, the druggability of FGFR3 has been demonstrated in cervical cancer *in vitro*, and now, clinical studies with pan-FGFR inhibitors in HNSCC patients are underway (32, 44, 45). Despite these encouraging findings, actual pre-clinical validation in terms of pharmacological inhibition of the FGF pathway in HPV^+^-HNSCC is still in its infancy. For research that is more mature, we must shift our focus to Her/ErBb receptors. EGFR has been a primary target for much of HNSCC drug discovery efforts, and a number of monoclonal antibodies and small molecules such as erlotinib and gefitinib have been developed. Single-target, small molecule tyrosine kinase inhibitors, however, have been found to be more toxic and less efficacious than the clinically approved cetuximab (46, 47). In addition to EGFR, other members of the Her/ErBb family may also be therapeutic targets. A study by Jasenka Mazibrada et al. found that the Her2 (ErBb2) protein is overexpressed in HPV^+^ patient samples relative to their HPV^-^ controls (48). A more extensive study by Netanya I Pollock et al. demonstrated that the protein levels of Her2 as well as Her3 (ErBb3) and the Her2/Her3 heterodimer were higher in HPV^+^-HNSCC tissue samples (21). Interestingly, HPV oncoproteins were shown to be involved in the regulation of Her3 (22, 43). Accordingly, a number of preclinical studies have tested and confirmed the therapeutic relevance of Her3 overexpression in HPV^+^-OPSCC. A Her3 antibody called MM-121/SAR256212 has been tested on cell lines and xenografts and showed molecular inhibition of Her3, Akt, mTOR activation and reduction in cell survival and tumor growth (49). In another study, use of both anti-Her3 siRNAs and monoclonal antibody (CDX3379/KTN3379) demonstrated good control of tumor growth in HPV^+^ PDXs (22). The feedback up-regulation of Her3 has also been confirmed as a route of resistance to PI3K inhibition, and dual use of anti-Her3 antibody improved the efficacy of PI3K inhibitors (43). In addition to monoclonal antibodies, multiple Her small molecule inhibitors have also been tested and shown to be effective in HPV+ OPSCC cells. Lapatinib, a dual EGFR/Her2 inhibitor, showed higher cytotoxicity in HPV^+^ cell lines (50). Afatanib, a Her1/Her2/Her4 inhibitor, has also been shown to have efficacy in cell lines (21). Clinically, both antibody therapy and small molecule tyrosine kinase inhibitors (TKIs) have been tested. The efficacy of CDX3379/KTN3379 is currently being evaluated in clinical trials. For Duligotuzumab (dual anti- EGFR and Her2) and Patritumab (anti-Her 3), results have shown mediocre efficacy (51, 52). For the TKIs, lapatinib and afatinib and the pan-Her inhibitor dacomitinib, no significant clinical benefits were observed (28, 44). Once again, there is a discord between pre-clinical findings and clinical observations for inhibitors of the PI3K pathway, for reasons that are not yet clear. What is clear, however, is that PI3K signaling in OPSCC is complex, and that targeting multiple PI3K pathway effectors at once may be more efficient than monotherapies. More importantly, various effectors of the PI3K pathway have been implicated in mediating resistance to cetuximab therapy (53–55). A recent trial found that HPV^+^-OPSCC patients carrying PI3KCA mutations have worse treatment responses and poorer prognosis than those without the mutations, and this is key given the subpar performance of cetuximab in de-escalation trials (56). All this shows that effective inhibition of the PI3K pathway in OPSCC is still somewhat of a black box, and that untangling the complex signaling to circumvent resistance mechanisms will be critical before the agents can be viable candidates for de-intensification. In addition, the success of PI3K inhibitors and potential integration into clinical use may hinge on their efficacy in combination with standard of care. The PI3K pathway limits the effect of RT and chemotherapies by promoting cell survival and DNA repair, and thus PI3K inhibitors could, in principle, potentiate the effect of such therapies. A number of emerging studies, both pre-clinical and clinical, indicate that this may be the case for some PI3K inhibitors in combination with cisplatin and radiotherapy (27, 57–61).

## Targeting the DNA Damage Response Pathways

Molecular targeted agents do not have to directly kill tumor cells or stop their growth to be considered effective. Such agents could also indirectly potentiate the effects of death-inducing agents such as DNA damaging therapies. Many DNA damaging agents are known to nick the DNA and cause single strand breaks (SSBs) and double strand breaks (DSBs) when applied to cells (62). Under normal conditions, the repair of these DNA lesions is triggered by a network of mechanisms (**Figure 2**) that sense and signal for repair of DNA breaks called the DNA damage response (DDR). SSBs are repaired by base excision repair (BER), and DSBs are repaired by either homologous recombination (HR) or non-homologous end joining (NHEJ), depending on the cell’s cycle phase (62). Having intact and efficient DNA repair mechanisms is therefore imperative for normal cells to maintain the integrity of their genome and survive (63, 64). In some cancers, defects in one or more of these DNA repair pathways exist, and the tumor cells must then rely on the remaining viable repair mechanisms to deal with breaks or stalled replication forks. Selective inhibition of this addiction to the rescue repair pathway(s) can generate synthetic lethality and profoundly sensitize the cancer cells to DNA damaging agents such as chemotherapy and ionizing radiation (63, 64). This strategy has led to robust clinical successes with PARP inhibitors in HR-deficient ovarian and breast cancers.

Importantly, examination of DNA repair pathways in HPV^+^-HNSCC has revealed that this cancer subtype harbors DSB repair defects (65–68). Specifically, HPV^+^-tumors seem to lack intact HR and NHEJ pathways (69–73). Different studies have independently demonstrated that the function of several key proteins in these repair pathways, such as BRCA1/2, 53BP1, Rad51, DNA-PKCs and TRIP12, may be impaired (69–72). In fact, the remarkable sensitivity of OPSCC to chemotherapy and radiation has been attributed to the existence of these repair defects (3, 74). In addition, data in the literature shows that HPV^+^-HNSCC cancers also exhibit an increase in activity of some components of the BER pathway, implying dependency on this back up pathway for DNA repair. For instance, BER-related genes such as PARP-1, DNA Polb, XRCC1, Lig I have been found to be up-regulated in a functionally meaningful way in HPV^+^-HNSCC cancers (70, 75). These findings lead to the proposition that DNA repair pathways can also be a therapeutic soft spot in OPSCC, which has been the impetus for the recent investigation of PARP inhibitors as potential radio-sensitizers. Recently, a PARP inhibitor called veliparib (ABT-888) has been found to be effective in increasing radio-sensitivity both in cells and xenograft models of HPV^+^ HNSCC (69, 75). Olaparib has also been tested in HPV^+^-HNSCC cell lines with encouraging results (70, 72, 76, 77). A third PAPR inhibitor, niraparib, has similarly led to radio-sensitizing effects in HPV^+^-HNSCC cells (78). Interestingly, niraparib also improved the relative biological effectiveness of protons by about 10% in HPV^+^ cells compared to 3% in HPV^-^ cells. PARP inhibitors have also been investigated in HPV^–^HNSCC and have shown some efficacy, even though HPV^–^HNSCC cancers are generally deemed HR-proficient. The molecular mechanisms behind the synergistic effects in HPV^–^HNSCC, HR-proficient cells are yet not clear (70, 74, 76, 78, 79). In addition to the PARP inhibitors, NHEJ pathway inhibitors such as those targeting DNA-PKcs may also present viable therapeutic options. The DNA-PK inhibitor, N7441, showed effectiveness in enhancing radio-sensitivity when tested in HPV^+^ cell lines (75). This developing evidence is outlining a path for clinical translation, and various clinical trials are underway to evaluate the benefits of DNA damage-targeted therapies alone and in combination with radiation and/or chemotherapy largely in recurrent, metastatic (R/M) disease (74) and to a lesser extent, LA HNSCC (**Table 1**).

In addition to the canonical DNA repair pathways, another emerging repair mechanism of translational relevance, the microhomology-mediated end joining mechanism (MMEJ), can also be considered a potential target (**Figure 2**). MMEJ, also known as alternative end-joining, is a third route utilized to repair DSBs, in addition to HR and NHEJ (73, 74). Studies show that cancer cells without HR and NHEJ up-regulate this error-prone DNA repair pathway to survive (75). Interestingly, like the BER, this pathway also relies on PARP-1 function, and inhibition of this pathway through PARP inhibitors may contribute to the observed PARP-directed synthetic lethality. In addition to PARP-1, DNA POLQ plays an essential role in MMEJ. Recent studies have demonstrated that inhibition of PolQ in DSB repair-deficient cancers, as in the case of PARP-1, also results in synthetic lethality (80). Notably, recent examination of genomic data and results from TCGA analysis have demonstrated that not only do HPV^+^-tumors show marked dependency on MMEJ to repair DSBs, but also that POLQ is up-regulated compared to HPV^-^ cancers (72, 81). These data suggest that future pharmacological intervention targeting PolQ alone or in combination with PARP-1 and DNA damaging agents may have promise as an additional therapeutic strategy for HPV^+^-HNSCC. Selective therapies targeting this protein are in development, and one group has found an inhibitor that specifically inhibits POLQ enzyme activity (80). Taken together, identification of inhibitors of DNA repair pathways and their successful integration into treatment cocktails as sensitizers to improve the therapeutic window of standard cytotoxic regimens may significantly contribute to de-intensification strategies.

## Targeting Cell Cycle Regulation

HPV^+^- and HPV^–^HNSCC tumors exhibit some functional equivalency in how they inactivate cell cycle regulators. HPV^+^ cancers express oncoproteins that accelerate the degradation of p53 and Rb through the ubiquitin proteasome system. In HPV^-^ cancers, mutations and promoter methylations affecting p53 and CDK2A (which encodes p16INK4a and p14) are common (1, 82, 83). These aberrations all impinge on the G1-S transition and progression of the cell cycle. Despite this commonality, the targetability of the differentially expressed proteins specific to each system is not always equivalent, and this impacts strategies for selective therapeutic inhibition. For instance, HPV- cancers often have gene copy gains in CCND1 (which encodes for cyclin D1) and amplifications and activating mutations of CDK4. Both events stabilize and activate the kinase activity of the CDK4/6 complex (82, 83). On the other hand, HPV^+^ cancers show amplification and overexpression of p16 and E2F1 (1, 82). These differences have been attributed to the distinct responsiveness of the two subtypes to standard treatments, and may also be potentially exploited for targeted therapy (3, 84). A case in point is the study by Gottgens EL et al. which targeted CDK4/6 and showed that Palbocyclib is effective in increasing the radio-sensitivity of HPV^-^ cells (85). Another CDK4 inhibitor, ryuvidine, has also recently shown efficacy in HPV- cancer cells (86). Palbocyclib and other CDK4/6 inhibitors are now being explored for efficacy alone or in combination with other therapies in clinical trials (28, 87). For HPV^+^-cancers, Gary C et al. demonstrated that an inhibitor of CDK1, 3, 5, 7 and 9 called roscovitine showed significant HPV-selectivity in both cell line and rodent xenograft models (88). Roscovitine has also been shown to increase the effectiveness of RT and chemotherapies in other cancers (89). In addition to palcocyclib and roscovitine, inhibitors of Chk1 and Wee1 are among other cell-cycle regulating agents that have been investigated pre-clinically. Specifically, inhibitors of Chk1 and Wee1 have been found to be effective as radio-sensitizers in HPV^+^ cells by blocking radiation-induced G2 arrest (86, 90–92). Some Chk1 and Wee1 inhibitors are also currently being tested in the clinic (74) (*also see* **Table 1**). In summary, more studies are needed to evaluate the potential of cell cycle regulators in the treatment of these cancers.

## Therapies Focused on p53 Restoration and Other HPV-Specific Targets

As previously noted, the E6 and E7 viral oncoproteins present in HPV^+^-cancers promote the degradation of p53 and Rb, respectively. The consequences of the inactivation of these tumor suppressors are multiple, including accelerated cell cycle progression, attenuation of cell cycle arrest and apoptosis induction. Similarly, mutations in p53 have been associated with inhibition of apoptosis, low response and resistance to radiation in HPV^–^HNSCC (93–95). In addition to p53, E6 also inactivates several other cellular substrates including Caspase 8 and Bak (**Figure 2**), both proteins involved in apoptotic signaling. Through these functions, HPV oncoproteins influence the growth and survival of tumors, as well as their response to treatment. Because E6 blocks both the extrinsic and intrinsic apoptotic pathways, reversing its effects in HPV^+^ tumors is predicted to enhance the efficacy of apoptosis-inducing agents such as RT and chemotherapy (96–99). Restoration of E6-targeted substrates such as p53 is therefore a reasonable therapeutic intervention.

Using a molecule called RITA that prevents E6 binding to p53, Zhao CY et al, have successfully rescued p53 in HPV^+^ cancer cells. In this study they showed that RITA transactivated p53, induced apoptosis and caused tumor regression in mouse xenografts (100). Xie et al. used a different strategy that employed the CH1 domain of p300 (an activator of p53) to competitively inhibit E6 binding to p300. This approach resulted in transactivation of p53 and inhibition of tumor growth in a NOD/SCID mouse model. A novel pharmacological small molecule mimic of the CH1 domain called CH1iB confirmed these results (101). RITA and CH1iB have also demonstrated the potential for p53 reactivation as a therapeutic strategy in combination with standard therapies in HPV-associated cancers (101–103). In our laboratory, we have screened small molecule libraries for specific inhibitors of E6 and have identified candidates that are selective for HPV^+^-cancers, including HNSCC cell lines. Our earlier efforts identified spinacine, a molecule that restored both p53 and Caspase 8 levels and significantly sensitized HPV^+^ but not HPV^-^ cells to chemotherapies such as CCDP (97). Recently, we have identified another inhibitor of E6, 30-hydroxygambogic acid (GA-OH), that rescued p53 function and induced apoptosis in an HPV^+^-selective manner (104). Not only did we show synergistic interactions between GA-OH and chemotherapy agents, we also observed radiosensitization in an HPV-dependent manner (unpublished). Other agents have been found to reduce the expression of HPV onco-proteins at the mRNA level, restoring p53 function and enhancing radiation sensitivity (105). More recently, an agent with a similar mode of action called Minnelide has been identified and been shown to inhibit tumor growth both *in vitro* and *in vivo* (106).

Another unique aspect of HPV^+^-cancers is their methylation profile signature (107). Although more extensive research is needed to fully validate the findings, examination of HPV^+^-cancers has revealed that HPV oncoproteins dysregulate activity of DNA methyltransferases, and that the genome of these cancer cells contains hypermethylated regions including promoters (19, 20, 107). Because of this unique epigenetic landscape and the role it plays in the expression of HPV proteins and cellular genes, Biktasova A et al. has explored the use of the global demethylation agent 5-azacytidine (5-aza) (108). 5-aza reduced E6/E7 expression, stabilized p53 and induced p53-depended apoptosis in HPV^+^-HNSCC cells. These results were recapitulated in xenograft mouse models, where they inhibited both growth and metastatic potential of cancer cells. More importantly, results from a window clinical trial confirmed these pre-clinical results in HPV^+^-HNSCC patients (108). These HPV-directed strategies are promising and have the potential to improve the therapeutic index of apoptosis inducers. However, 5-aza is currently the only HPV-specific agent that has thus far progressed to patients (**Table 1**); the rest of the aforementioned compounds remain in pre-clinical development and are undergoing further experimental validation.

## Targeting the Tumor Microenvironment of HPV^+-^HNSCC

Immunotherapy, particularly the use of immune checkpoint inhibitors (ICIs), is another approach that has potential as a candidate for de-intensification. Although immunotherapies do not directly target driver oncogenic events in tumor cells, their effect on non-cancerous, immune cells in the tumor nest can hugely impact on tumor control and response to therapy. Various ICIs including anti-PD1, anti-PDL1 and CTLA-4 antibodies have been tested for the treatment of HNSCC and two ICIs, nivolumab and pembrolizumab, both anti-PD-1 antibodies, were granted FDA approval for platinum refractory patients in 2016. More recently, pembrolizumab has been approved as a first-line therapy alone or in combination with platinum/fluorouracil based on PD-L1 expression for patients with R/M disease. This progress in the last 5 years is significant and remarkable given that immune function is depleted in R/M tumors compared to earlier stages such as LA disease. Additionally, these immunotherapies have also shown to be more tolerable than chemotherapy, improving both OS and QoL (109). These factors coupled with a deeper understanding of the immune context of HNSCC in the future supports the notion that ICIs make good candidates for de-intensification for LA HPV-related disease.

A close look at HPV^+^- and HPV^–^HNSCC confirms that these two entities are immunologically distinct diseases. Part of this distinction comes from their differences in mutational profiles. HPV-HNSCC has a relatively high tumor mutation burden (TMB) and a wider spectrum of mutations (109). A high TMB normally results in more non-silent mutations as well as neo-epitopes that elicit an immune response (13). The tumor mutational load in HPV^+^ tumors, on the other hand, is relatively more modest, noting that these tumors also express viral proteins. HPV proteins have no human homologues, and their antigenicity has been proven to be quite robust (13, 110). Also, HPV^+^-HNSCC primarily originates in the more lymphoid-dense regions of the head and neck. These differences certainly influence the unique recruitment of immune cells and the resulting landscape seen in the two cancers (84). Indeed, research data show that HPV^+^-HNSCC is richer and more diverse immunologically in terms of both gene expression and cellular composition. Immune subtyping of HPV^+^ and HPV^-^ cancers based on RNAseq by Kim MH et al. showed that HPV^+^-OPSCC, and not its counterpart, falls into the most immune-rich category (111). This category was also associated with infiltration of CD8^+^ PD-1^+^ T cells and type I macrophages, and correlated well with favorable response to ICIs. Another study corroborated this observation, and found that HPV^+^ is characterized by high expression levels of several immune genes including CD8, CD56, ICOS, LAG3, HLA-DR (13). Again, these tumors were infiltrated with CD8^+^ T and NK cells and enriched in the immune “inflamed”/mesenchymal (IMS) subgroup (13). Interestingly, in addition to this “inflamed” phenotype, HPV^+^-HNSCC is also characterized by the presence of CD4^+^ CD25^+^ Tregs and high expression of PD-1, CTLA-4 and TIM-3 on the T cells (13). Moreover, the HPV^+^-HNSCC tumor cells themselves also express higher levels of the PD-1 ligand, PD-L1 (3). In contrast, HPV^–^HNSCC have significantly lower levels of immune infiltrates and relatively lower numbers of CD8^+^ T cell and exhaustion markers, despite their higher mutational burden. In fact, among infiltrated tumors, a pan-cancer analysis showed that HPV^+^-HNSCC had the highest median of immune infiltrates (112). Interestingly, the presence of immune-regulatory cells such as Tregs and exhaustion markers in HPV^+^-HNSCC corresponded well with clinical outcomes (13, 111, 113). In contrast, in HPV^–^HNSCC, an immune regulatory character was detrimental to patient prognosis. Only high levels of CD8 T cells and high TMB correlated positively with prognosis for HPV^-^ patients (13). However, there is still no one concrete way to predict patient response to ICIs in HNSCC.

Along those lines, evidence suggests that it is too early to safely conclude whether HPV status is associated with superior outcomes following treatment with ICIs. Indeed, a few studies have reported that there is no discrimination between patients by HPV status (113, 114). On the other hand, more studies have reported the opposite. For instance, in the clinical trial (Keynote-012) that evaluated pembrolizumab in R/M HNSCC patients revealed that even though ORR was 18%, analysis by subgroup revealed that the response rate was 25% versus 14% in HPV^+^ and HPV^–^HNSCC patients, respectively (13, 115, 116). In a different clinical trial with durvalumab, an international, multi-institutional, single-arm study, NCT02207530, revealed an ORR of 16.2%, which translated into 29.4% in HPV^+^ and 10.8% in HPV^-^ patients when separated by HPV status (115). A more systematic literature review of ICI response in HNSCC by Ghanizada M et al. found that in 4 out of 5 studies they assessed, OS or PFS were superior in HPV^+^ than in HPV^-^ patients (115–117). Additional and larger studies are thus needed in the future to conclusively define the nature of any association between HPV and ICI response, so as to guide treatment decisions for HPV^+^-HNSCC patients. As the research on this continues, studies exploring an exciting alternative strategy involving the combination of ICIs with other traditional treatments such as radiation, with or without de-escalation intent are already underway (13). There is good rationale for these combination studies, given that radiation can promote inflammation, induce immunogenic cell death and enhance antigen presentation for an enhanced anti-tumor response (110). Chemotherapy can also release neoantigens and deplete immunosuppressive cells (110). Harnessing the positive effects of radio- and chemotherapy can therefore increase the proportion of responders and amplitude of response to ICIs and help with de-escalation endeavors. To that end, a number of clinical trials have incorporated ICIs in combination with radiation, chemotherapy or experimental targeted therapies for evaluation in LA HNSCC (118–123). Although not all of these clinical trials were designed with de-escalation intent, successful combination of ICIs with radiation in LA HNSCC can still be a boon for the HPV^+^ cohort if it comes with less toxicity compared to platinum-based CRT. As of now, it is still too early to tell what conclusions these trials will ultimately support. So far, the results have been negative, as evidenced by the JAVELIN head-and-neck 100 Phase III trial that failed to improve OS with avelumab as compared to cisplatin/radiation therapy (124). The same can be said about the GORTEC 2015-01 PembroRad trial, which failed to improve locoregional control over SOC using pembrolizumab plus radiation (125). However, these are just two of the many ongoing trials involving LA HNSCC (118–123). More importantly, some recent trials, albeit fewer in comparison, are now evaluating ICIs combinations specifically in LA, HPV^+^ OPSCC with and without reductions in the dosage of radiation (**Table 1**). All these ongoing efforts are exciting new developments and the field eagerly awaits the results with the hope that new findings will change the trajectory for future HPV^+^ patients.

## Conclusions

Treatment guidelines for HNSCC are currently evolving, particularly for HPV^+^-HNSCC. Future paradigms may incorporate a switch from escalated therapies to less toxic, de-intensified CRT regimens for locally advanced disease, given the amplification of toxicities by chemotherapy-based radio-sensitizers (**Figure 1**). In a bid to uncover novel, more potent and less toxic therapies, a better understanding of the molecular landscape of HNSCC subtypes is under development. Targetable alterations, albeit few, have been found. Despite the noted limitations, prospects for therapeutic intervention using agents that target the tumor immune micro-environment, particularly the immune checkpoints, are promising, and such agents may be integrated into future de-escalation regimens. Other targets, such as the PI3K or p53 restoration pathways, require additional research to validate these approaches pre-clinically. In particular, more extensive pre-clinical research is needed to validate the functional consequence and clinical utility of inhibiting these targets, as well as any associated compensatory activation signaling. Importantly, it is not only comprehensive and convincing pre-clinical validation that is required to increase the chances of clinical success. Design of clinical trials based on patient risk-stratification and molecular selection will also be key. Emerging evidence shows that clinical factors and dynamic biomarkers such as circulating HPV DNA may determine which patients obtain therapeutic benefit in clinical studies (10, 126). In addition, genomic biomarkers such as PI3K and p53-associated mutations may also have predictive power in terms of prognosis. Ongoing research will continue to pre-clinically substantiate efficacy of targeted therapies and the associated resistance mechanisms, and refine those findings for clinical leverage.

## Author Contributions

LC wrote the manuscript. PD-H edited and supervised the manuscript. All authors contributed to the article and approved the submitted version.

## Funding

Funds supporting the publication of this work were provided by Loma Linda University and the Fletcher Jones Foundation.

## Conflict of Interest

The authors declare that the research was conducted in the absence of any commercial or financial relationships that could be construed as a potential conflict of interest.

## Publisher’s Note

All claims expressed in this article are solely those of the authors and do not necessarily represent those of their affiliated organizations, or those of the publisher, the editors and the reviewers. Any product that may be evaluated in this article, or claim that may be made by its manufacturer, is not guaranteed or endorsed by the publisher.
